# Designing minimalist membrane proteins

**DOI:** 10.1042/BST20190170

**Published:** 2019-10-11

**Authors:** Paul Curnow

**Affiliations:** 1 School of Biochemistry, University of Bristol, Bristol, U.K.; 2 BrisSynBio, Life Sciences Building, Tyndall Avenue, Bristol, U.K.

**Keywords:** membrane proteins, protein design, synthetic biology

## Abstract

The construction of artificial membrane proteins from first principles is of fundamental interest and holds considerable promise for new biotechnologies. This review considers the potential advantages of adopting a strictly minimalist approach to the process of membrane protein design. As well as the practical benefits of miniaturisation and simplicity for understanding sequence-structure-function relationships, minimalism should also support the abstract conceptualisation of membrane proteins as modular components for synthetic biology. These ideas are illustrated with selected examples that focus upon α-helical membrane proteins, and which demonstrate how such minimalist membrane proteins might be integrated into living biosystems.

## Why design membrane proteins?

It is estimated that up to 30% of all proteins are integral membrane proteins [1], with at least one part of the protein sequence passing through a lipid bilayer membrane. This abundance reflects the universal role of such proteins in essential life processes such as signalling, solute transport, bioenergetics and much more. Developing a basic understanding of membrane protein biosynthesis, trafficking, insertion, folding, and assembly continues to attract considerable attention [2–7]. There is also now a growing interest in using membrane proteins in the emerging field of synthetic biology (e.g.[8–11]). In this minireview, we discuss the prospects of designing artificial membrane proteins from scratch — ‘*de novo*’ — as a particular way to explore the fundamental principles of membrane biology and to realise new applications in synthetic biosystems.

The motivations for membrane protein design are much the same as those put forward for ‘water-soluble’ proteins, which have recently been discussed in several excellent reviews and perspectives [12–19]. We do not intend to reproduce these arguments in full here, but they can be broadly and briefly summed up as follows. Natural proteins are intricate and complex, which can obscure the core physical principles underlying their structure and function. Building simplified model proteins can, therefore, be a useful way to cut through this complexity and understand the fundamental connections between sequence, structure and function. Another motivation for the designer is that natural selection has not had sufficient time to sample every possible combination of amino acids. Hence there are a large number of protein sequences (and by extension, structures and functions) that have never yet occurred in the natural world. *De novo* design allows us to survey the full scope of protein sequence and structural space, and to see whether artificial constructs could replace natural proteins or have useful non-natural functions. Exciting innovations in the field take advantage of new enabling technologies such as gene synthesis, automation and recombinant engineering as well as the improved accessibility, lower cost and greater sophistication of established techniques including peptide synthesis, mass spectrometry, NMR and computation.

In the case of integral membrane proteins, there are at least two additional motivations for the prospective designer. The most obvious stimulus is that our understanding of membrane proteins is now being revolutionised by a dramatic increase in the number of high-resolution structures available - although at the time of writing this still only accounts for <2% of all structures deposited in the protein databank [20]. This progress in structural biology provides a rich new source of information for design; for example, as discussed below, analysing known structures can identify general sequence motifs involved in transmembrane helix packing [21,22]. A second impetus is that the design of membrane proteins has been less well-explored compared with their water-soluble counterparts and so there remains much to learn. One aspect of this is that membrane proteins have a notorious reputation for being difficult to work with. Low expression levels, misfolding and non-specific aggregation can often frustrate efforts to apply biophysical methods to membrane proteins. This can make it hard to judge the success of the design process. However, both the knowledge base and practical toolkit around expressing, purifying and reconstituting membrane proteins continue to rapidly mature, which now substantially improves the chances of overcoming these barriers for *de novo* membrane proteins [23–29].

Given that there seem to be good reasons to engage in membrane protein design, how should one proceed? A number of different approaches are possible, and the field embraces a swathe of creative computational and knowledge-based strategies that account for some notable recent successes [30–33]. Here, we will discuss ascetic minimalism as one particular tactic for the design of *de novo* integral membrane proteins. We suggest that the stage is set for the development of genetically encoded, low-complexity proteins that can provide new insights into membrane biogenesis and can be harnessed to introduce novel functions into living cells.

## A manifesto for minimal membrane proteins

**Figure 1.**
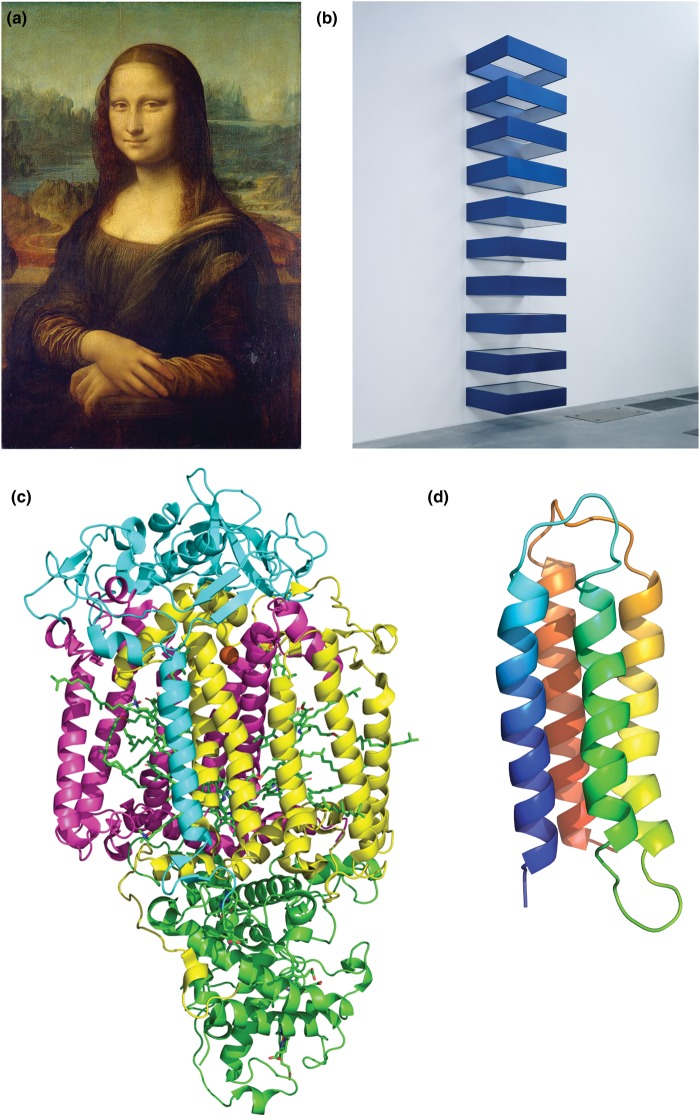
The concept of minimalism in protein design. (**a** and **b**) Minimalist art rejects traditional representations of the natural world, such as landscapes and portraits, in favour of abstract, serial, and repetitive 3D forms. (**c** and **d**) By analogy, minimalist membrane proteins do not attempt to recreate the complex sequences and structures of natural proteins but instead provide modular biocompatible objects with some specific properties. (**a**) *La Gioconda* (‘*The Mona Lisa*') Leonardo Da Vinci (1452–1519) from https://en.wikipedia.org/wiki/Mona_Lisa. (**b**) *Untitled*, 1990 Donald Judd (1928–1994) © Judd Foundation/ARS, NY and DACS, London 2019. From the Tate Images Collection © Tate, London 2019. (**c**) Photosynthetic reaction centre from *R. viridis*, PDB ID 1PRC [102]. (**d**) Molecular model of a minimal *de novo* membrane protein (not to scale with (**c**)) adapted from ref. [82] (https://creativecommons.org/licenses/by/4.0/).

The concept of minimalism emerged from the art world in the 1960s and has since been adopted by theatre, choreography, music, architecture, and other spheres. Although minimalism invokes simplicity and reductionism, it is more than just a paring-back of superfluous elements [34]. The original minimalist artworks are three-dimensional objects that lack any ornament or obvious makers’ craft. They are modular and repetitive, and the methods and materials of construction are obvious. They are geometric and deliberately eschew any reference to the organic or natural. Fundamental to minimalist art is that the work contains little illusion or allusion, ensuring that the meaning of the piece is constructed by the viewer rather than the originator (Figure 1). By analogy, minimalist *de novo* membrane proteins should have little or no sequence similarity to natural proteins. They should be of the lowest-possible sequence complexity, using the fewest available amino acids to achieve their form and function, and be modular at the level of either sequence or structure. They ought to stand alone and not be fusions to other, natural, proteins. And they ought to ideally be genetically encoded, so that they might eventually become part of cell physiology and be susceptible to laboratory evolution.

What is the case for such austere minimalism in membrane protein design? There should be some straightforward advantages to diminution and simplification, which have been recognised previously [35,36]. Minimal membrane proteins should allow us to identify the most basic sequence requirements for particular behaviours and functions. Such elementary proteins could be more predictable, tractable, pliable, adaptable and robust than many natural membrane proteins. When recombinantly expressed one might expect minimalist proteins to impose a relatively low demand for cellular resources and to be highly orthogonal to the host cell, minimising the metabolic load on the host and reducing the potential for unwanted interference with natural pathways. The lower the complexity of the protein sequence, the easier it should be to understand the impact of individual mutations on the protein as a whole. In many ways, this seems like a natural progression of the widespread and influential use of minimal transmembrane segments to probe multiple aspects of membrane protein biophysics [37–39]. A full consideration of this extensive body of work lies outside the scope of the current short review, but examples include the study of protein–lipid interactions [40,41], protein–protein interactions [42–45] and the biogenesis of transmembrane helices [46,47].

Minimal proteins could provide other benefits to the field of synthetic biology. The unifying vision of synthetic biology is the *artificial cell*; a man-made complex system that diverges to a greater or lesser degree from extant modern life [48–50]. Realising this ambitious goal in its fullest sense requires not just an artificial *genome*, but an artificial *proteome*. Minimal *de novo* proteins could provide modular ‘building blocks’ for assembling such a proteome, and will be useful in allowing the size of the synthetic genome to be as small as possible. But there are also implications of minimalism that go beyond this.

One important part of synthetic biology is abstraction. With regards to proteins, this requires that they are seen less as the compelling products of natural history and more as engineering components: durable, reliable, consistent and interchangeable parts to be adopted or discarded as the situation demands. But natural proteins come with an origin story — a narrative constructed around their discovery and study. We ought to recognise that these narratives and contexts make it difficult to really see proteins in the same light as a house brick or hose-clip, however much we might think otherwise. For example, one of the cornerstones of synthetic biology research is the green fluorescent protein (GFP). Scientists and schoolchildren alike cherish the story of GFP; how a seemingly unpromising source, the jellyfish, gave rise to a revolutionary technology that has transformed the molecular biosciences. This means that like many natural proteins GFP remains indelibly associated with certain people, places, and times. Truly artificial proteins circumvent this issue since they have no such narrative constructed around them, but instead are deliberately utilitarian. It seems reasonable to suggest that the simpler such *de novo* sequences are, and the less they echo their natural counterparts, the easier it will be to think of them in the abstract. In this spirit, minimalism appears to be an important, productive and liberating area for protein design.

## How to realise minimalism in membrane protein design?

The most obvious targets for the minimal design of membrane proteins are transmembrane α-helical bundles, and this will be the focus of the discussion below. Nearly all of the natural proteins in cell plasma membranes feature α-helical secondary structure and these integral membrane proteins are responsible for a wealth of different cellular functions. The remainder of membrane proteins are β-barrels, which occur in the outer membranes of bacteria, mitochondria and chloroplasts and have received less attention in terms of engineering and design [51,52].

### Amino acid sequences

The interior of a lipid bilayer membrane is hydrophobic. It is energetically favourable for hydrophobic amino acid sidechains to partition into this environment, but energetically unfavourable for exposed peptide bonds to do the same. These competing effects are resolved by the spontaneous formation of a transmembrane α-helix, which exposes the hydrophobic sidechains to the bilayer interior and fully satisfies backbone hydrogen bonding. This means that virtually any sufficiently hydrophobic sequence of ∼18–26 amino acids can form transmembrane α-helices that are long enough to span a biological lipid bilayer [4]. This suggests that it will be relatively easy to reduce the complexity of transmembrane segments while retaining secondary structure, and indeed the lipophillic domains of natural helical membrane proteins are already built from a somewhat restricted palette of amino acids. Slightly over half of all the amino acids in biological transmembrane helices are the hydrophobic residues Leu, Ile, Val, Phe and Ala, with Leu being most prevalent [53–55]. Others, especially the charged residues Glu, Arg, Lys and Asp, are relatively rare (although certainly can be found, and Lys and Arg in particular can be accommodated by their ‘snorkelling’ into the lipid headgroup region [56,57]). The primary consideration then is not whether a minimal sequence based on one or more of the common hydrophobic amino acids will form a transmembrane helix, since we may assume that it will do so, but how these helices will interact with the membrane and with each other.

### Interactions of transmembrane helices

**Figure 2.**
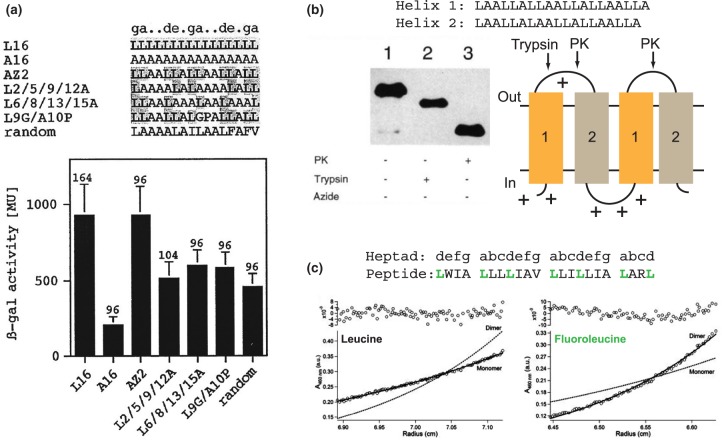
Some approaches in the minimalist design of membrane sequences. (**a**) A transcriptional activation assay shows that polyleucine sequences self-assemble *in vivo*. Mutational analysis confirms the importance of leucine at heptad positions *a* and *d*, suggesting a leucine zipper interface. Republished with permission of The American Society for Biochemistry and Molecular Biology from Gurezka et al. [44] © 1999. Permission conveyed through Copyright Clearance Center, Inc. (**b**) Topological control of a recombinant minimal membrane protein. Two slightly different sequences for minimal TM helices are used to assemble a four-helix construct as shown, and charged residues are introduced at the N-terminus and intracellular loop to control topology according to the ‘positive inside’ rule. Topology within the *E. coli* inner membrane is assessed by proteolysis of the exposed periplasmic loops, which leads to the predictable differences in gel migration shown. *PK*, Proteinase K. Left panel adapted by permission from Springer Nature. Whitley et al. [81] © 1994. (**c**) Introducing hexafluoroleucine at heptad positions *a* and *d* encourages dimerisation of a minimal peptide as determined by equilibrium analytical ultracentrifgation. Adapted with permission from Bilgiçer and Kumar [86]. © 2004 National Academy of Sciences, U.S.A.

The membrane milieu impacts the type of interactions that can drive helix association. In the low dielectric of the membrane interior, sidechain hydrogen bonding can be a means of impelling helix association [43,45]. However, it appears that for many natural membrane proteins a key driving force is Van der Waals interactions. These forces are optimised through shape complementarity and sidechain geometry that allow the close approach of transmembrane helices [58]. In some cases, this also allows for the formation of interhelical Cα-H···O hydrogen bonds that likely influence both stability and packing specificity [59,60]. Close packing can be successfully accomplished even with a small subset of hydrophobic amino acids, probably supported by a lipophobic effect that favours protein–protein over protein–lipid interactions. For example, transmembrane polyleucine helices can self-associate via a ‘knobs-into-holes’ leucine zipper interaction also found in natural proteins (Figure 2a), and this is maintained when Leu is replaced at the helix interface by other hydrophobic amino acids [44,61]. It does seem that natural membrane proteins adopt a relatively limited range of helix–helix packing orientations. About 30% of natural antiparallel helical pairs exploit an Ala-coil motif to pack with a slight left-handed crossing angle [22]. In this motif, small amino acid sidechains (typically Gly, Ala or Ser) occupy positions *a* and *d* or *e* of the helical heptad to allow close packing via sidechain interdigitation. A frequent feature of both parallel and antiparallel right-handed pairwise packing interactions is the well-known small-xxx-small motif, where small residues occur at *i* and *i* + *4* sequence positions [22,55,59,62]. These small residues are often flanked by the β-branched sidechains Val and Ile, and it was suggested that the limited rotational freedom of these sidechains reduces the entropic cost of helix association [55]. These analyses were recently extended from helical pairs to consider helical trimers, which Feng & Barth [21] recognised as the most basic structural unit in natural membrane proteins. Just over half of these helical trimers could be sorted into one of six major categories. They identified 13 common sequence motifs that are able to optimise sidechain Van der Waals interactions as well as interhelical backbone–backbone and backbone–sidechain hydrogen bonds. Many of these motifs featured large hydrophobic residues at the helical interfaces. Although such simple sequence motifs are only one aspect of membrane protein folding [63], the examples above certainly describe features of helix packing in natural proteins that can be incorporated into minimal designs.

### Topology and biogenesis

Polytopic (or multipass) membrane proteins contain multiple helices that thread back-and-forth across the membrane and are connected by extramembrane loops. Establishing and maintaining the topology of these helices across the membrane is more complex than might have previously been thought [64–66], but a few general principles can be distilled. In bacterial systems, the major pathway for biogenesis of polytopic proteins involves the first TM helix acting as the membrane localisation sequence, also known as a signal-anchor. This engages with the signal recognition particle to target the nascent polypeptide to the cytoplasmic membrane and supports insertion via the Sec translocon. Protein topogenesis then has a close correlation with the character of the extramembrane loops, with cytoplasmic loops being enriched in positively charged amino acids. This is the ‘positive inside’ rule [67,68], which has been experimentally verified many times and can be exploited to deliberately dictate topology [69,70]. Another notable feature of natural proteins is the statistical preference for Trp and Tyr residues in the lipid headgroup region, which appear to be important as ‘anchors’ that control helical positioning and dynamics [40,71–74]. In the popular recombinant host *Escherichia coli*, about 80% of all membrane proteins have a topology that results in their C-terminus residing in the cytoplasm, and probably 60% have both their N- and C-termini within the cytoplasm [75]. A similar orientation bias was also found in the model eukaryote *Saccharomyces cerevisiae* [76]. Hence when contemplating the transmembrane topology of multipass *de novo* membrane proteins, locating both termini in the cytoplasm seems like a sensible design choice.

### Polyleucine as a minimal scaffold

Considering all of the above, polyleucine emerges as an appropriate starting point for the design of minimal α-helical membrane proteins. Leu has a high helical propensity, is the most common amino acid in natural transmembrane helices, and can engage in both protein–protein and protein–lipid interactions. Polyleucine is already established as a suitable and neutral ‘host’ for known motifs than can drive helical association. PolyLeu helices can be tolerated by biological systems and the topology of these helices can be controlled by the charge distribution in the flanking extramembrane domains.

## Minimal design: from concept to reality

**Figure 3.**
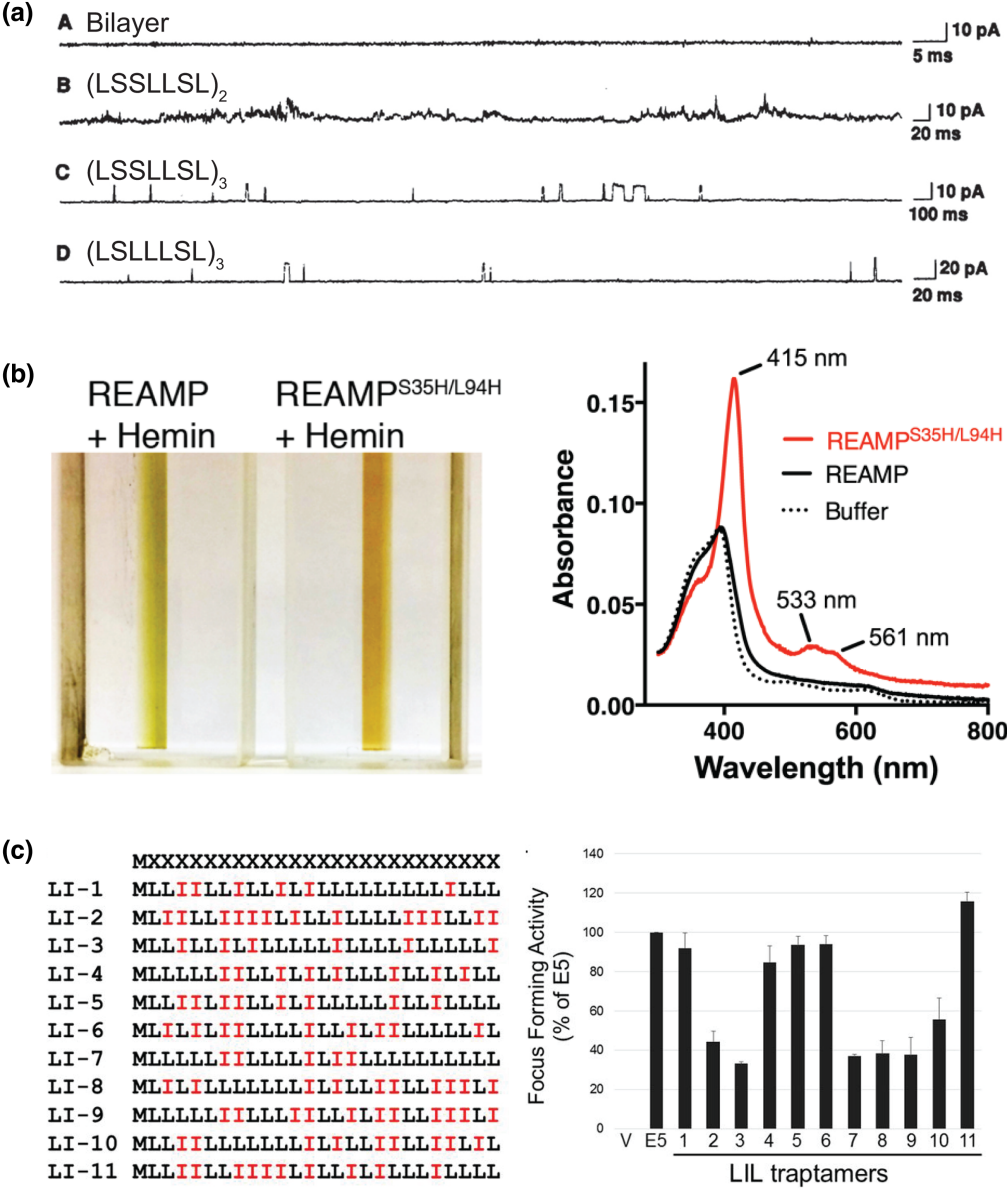
Examples of functionality in minimal membrane proteins. (**a**) Electrophysiology of synthetic lipid bilayers containing minimal ion channels. Panel *A* shows a negative control without any peptide; Panel *B* is control using a short peptide that cannot span the bilayer; Panel *C* is peptide with sequence (LSSLLSL)_3_; and Panel *D* is (LSLLLSL)_3_. From Lear et al. [80]. Reprinted with permission from AAAS, adapted with permission of the authors. (**b**) A minimal 4-helix bundle (‘REAMP'), in which each helix has the sequence LLLLSGLGLLLLSLLGLLLLS, can be induced to bind a haem cofactor via a bis-Histidine site. The reddish-brown colour and the spectroscopic fingerprint of the oxidised hemoprotein are both reminiscent of natural cytochromes. Adapted from ref. [82] https://creativecommons.org/licenses/by/4.0/. (**c**) A series of minimal single TM helices — termed ‘LIL proteins’ — interact with the PDGF-β receptor and transform mammalian cells. The activity of each sequence LI1–11 is compared with a natural activator, the small oncoprotein E5. Adapted with permission from Heim et al. [99].

The ideas outlined above can now be illustrated with some key examples of success in producing minimal membrane proteins. The first example seems to be from Goodall & Urry, who showed in 1973 that synthetic peptides built around an AAG repeat motif (e.g. (AAG)_4_) could form conductive ion channels across artificial membranes [77]. Subsequently, Kennedy et al. [78] extended this observation to polypeptides of various lengths containing the tetrad repeat motif LSLG. Peptides (LSLG)_12_ or an *N-*formyl derivative of (LSLG)_6_ also formed conductive channels that were proposed to arise from individual β-helices similar to gramicidin. However, a detailed molecular understanding was obviously difficult at that time [79]. A major leap forward was taken in a landmark paper from DeGrado and colleagues in 1988 [35,80]. This described the rational design of minimal peptides composed only of Ser and Leu that could recapitulate the dimensions and properties of individual α-helices found within natural ion channels. Two heptad repeat motifs were chosen for this work. The first of these motifs, LSSLLSL, was repeated in triplicate to give peptide (LSSLLSL)_3_. The expectation was that these peptides would self-assemble, with the Ser residues generating a polar helical face that would be sequestered from the bilayer interior to form an aqueous pore. The (LSSLLSL)_3_ peptide formed relatively permissive cation channels in synthetic lipid bilayers, with a pore size of ∼8 Å and conductance properties reminiscent of natural channel proteins (Figure 3a). Substituting Leu for Ser in the repeat motif to give peptide (LSLLLSL)_3_ produced channels with markedly different properties. These were apparently highly selective with much smaller pore size, being only permeable to protons. Computational models suggested that both peptides could assemble into parallel helical bundles with tightly packed helical interfaces driven by interdigitation of the Leu sidechains. However, (LSSLLSL)_3_ was likely to be a hexamer and (LSLLLSL)_3_ probably a trimer or tetramer. Both of these different tertiary structures allowed the Ser residues to be accommodated in the polar channel interior.

The minimalist approach was also exemplified by work from von Heijne and colleagues [81]. The sequence LAALLALLAALLALLAALLA was designed with the expectation that this would form ‘Janus’-like α-helices, with one face of the helix being Leu and the other face being Ala. Constructs comprising one, two or four successive TM helices were successfully biosynthesised from a recombinant plasmid (Figure 2b). These *de novo* proteins were localised to the *E. coli* membrane and the transmembrane topology was controlled by adjusting the charge in the interconnecting loops. These highly orthogonal constructs were easily accommodated by the cell — for example their expression did not inhibit cell growth.

Our own work [82] has built upon this by expressing a different minimal sequence into cellular membranes and then exploring the purification and characterisation of this *de novo* protein. Our design is an antiparallel four-helix bundle in which helices of sequence LLLLSGLGLLLLSLLGLLLLS are connected by short loops to give a N_in_/C_in_ topology. This bioinspired design was an abstracted version of the consensus sequence of the SMR group of small transporters. It could be expressed with the expected topology in the *E. coli* membrane and was surprisingly tractable to further study. The synthetic protein was purified in the gentle maltoside detergents that are widely used in membrane biochemistry and shown to be stable and monodisperse *in vitro*. The sequence was only designed for expression and did not specify any tertiary packing interactions, and so unsurprisingly appeared to be a dynamic molten-globule [82]. Goparaju and colleagues also showed that a minimal four-helix transmembrane bundle, with each helix built around subtle variations of polyLeu/Ala, could be recovered in detergents for *in vitro* analysis after being deliberately expressed into cellular inclusion bodies [83].

How easy is it to obtain well-defined structures from minimal sequences? A recent study demonstrated that Van der Waals packing interactions are sufficient to strongly constrain the structure of minimal membrane proteins, although the packing requirements are rather precise [33]. Inspired by the repeating LxxIxxx heptad packing motif (register *abcdefg*) observed in phospholamban, the authors computationally designed and chemically synthesised transmembrane peptides that assembled into tightly packed homopentamers. This was driven entirely by pairwise nonpolar packing interactions between neighbouring helices. These interactions could be very sensitive to mutation. For example, even a conservative Leu to Ile mutation at one *g* position was sufficient to disrupt packing and abolish oligomerisation. This provides substantial encouragement that low-complexity sequences could pack well enough to form well-defined structures, but that this will require judicious placement of residues at key positions.

An interesting alternative is to exploit the properties of non-natural amino acids. With regards to membrane proteins, fluorinated amino acid sidechains should favour protein–protein interactions over protein–lipid interactions [84,85]. This idea was validated by a study showing that low-complexity hydrophobic peptides containing hexafluoroleucine at the *a* and *d* heptad positions could assemble into either stable helical dimers or probable tetramers (Figure 2c), with the oligomerisation state depending on the presence of a single Asn in the membrane core [86,87].

Overall then, two key themes emerge. The first is that minimal membrane proteins are biosynthetically accessible — that they can be produced in cell systems and targeted to cellular membranes. The second is that minimal sequences could potentially form robust and well-defined structures by exploiting or mimicking the helical packing ‘rules' observed in natural proteins.

## Functional minimal membrane proteins

Once minimal sequences are identified, how can these be turned to some useful function? As discussed above, some minimal proteins can clearly form channels and pores (Figure 3a). In the rest of this review, we look at other means of functionalising the inert scaffold of a minimal membrane protein.

### Cofactor binding

The binding of porphyrins and related tetrapyrroles is an obvious target for introducing function into minimal membrane proteins. This is because these compounds are hydrophobic, so will spontaneously partition into lipid membranes and are good interaction partners for transmembrane segments. Natural porphoproteins are widespread and very well-studied, and the principles governing their structure and function are established. Metallated porphoproteins, particularly those containing haem, have a number of potentially interesting applications in electron transfer, gas binding and redox (bio)chemistry. Those containing chlorophylls can be photoexcited. Cofactor binding can rigidify otherwise dynamic *de novo* proteins [88,89] and indeed some structural flexibility in the apoprotein is probably desirable to support the interaction [90]. To this end, most studies with redesigned or *de novo* proteins have focussed upon the binding of haem. A popular strategy is to co-ordinate the haem iron with the imidazole sidechain of histidine, a binding mode that predominates in natural hemoproteins.

Glycophorin A is a natural single-pass membrane protein in which the membrane domain forms a homodimer. This was used as the basis for a *de novo* helical dimer that could bind haem via bis-Histidine ligation with apparent *K_d_* in the low μM range [91]. The subsequent hemoprotein was catalytically active as a peroxidase, with a redox potential of −128 mV. A single amino acid substitution was sufficient to both improve the binding affinity and adjust the haem redox potential by −44 mV [92]. Similar results emerged from the redesign of a natural helical peptide to adopt the Ala-coil motif [93]. This work generated a tetrameric dihaem membrane protein, which is of interest since two haems are needed to transfer electrons across a lipid bilayer. Korendovych and colleagues used computational design to develop a *de novo* four-helix bundle that also exploited the Ala-coil motif, and showed that this chemically synthesised peptide could co-ordinate two iron diphenylporphyrins via bis-His geometry in both detergent micelles and artificial membranes [94]. The redox potential of the two bound porphyrins was separated by 71 mV, being −97 and −168 mV. This splitting of the redox potential is typical for porphyrins that are in close proximity and could potentially facilitate biological electron transfer. The membrane segments of designs that blended water-soluble and hydrophobic regions were also found to bind haem and bacteriochlorophyll [95,96]. With regards to minimal proteins, Goparaju et al. [83] showed that four-helix bundles built from low-complexity Leu/Ala sequences could bind multiple haems as well as the photoactive complex zinc protoporphyrin IX via Histidine ligation. Bound haems had distinct redox potentials, and simultaneously binding both Fe- and Zn-substituted porphyrins allowed electron transfer between the two cofactors. Our own work has also shown that genetically encoded minimal proteins could be induced to bind a single haem *in vitro* (Figure 3b) with little modification other than introducing coordinating histidines [82]. These proteins had a redox potential of −101 mV, a shift in the haem potential of +32 mV from a hydrophobic non-protein environment, and nascent peroxidase activity that demonstrated the potential for developing these sequences for redox biochemistry and electron transfer.

It will be fascinating now to try and translate this understanding into living systems to influence cell physiology. This will require cofactor binding *in vivo* as well as the deliberate ‘tuning’ of redox potentials for specific applications. To our knowledge cofactor binding within a cellular membrane has not yet been achieved for *de novo* integral membrane proteins, although there is precedent for this from some soluble designs [97]. The biochemical pathways underlying cofactor synthesis are well-characterised, and so metabolic engineering to encourage such cofactor loading is plausible.

### Protein–protein interactions

Another potential application is to use minimal proteins to disrupt natural protein–protein contacts within the membrane, which are essential for the assembly of functional membrane complexes. This has already been achieved with more complex *de novo* peptides [98]. A recent report showed that small proteins comprising a single transmembrane domain consisting of only Leu and Ile (termed LIL proteins; Figure 3c) could be recombinantly expressed in mammalian cell lines [99]. Through library screening, specific sequence variants were identified that could influence cell biology by interacting with, and so activating, the TM domain of platelet-derived growth factor β receptor (PDGFβR) to initiate a signalling cascade. This remarkable result suggests that even proteins with elementary amino acid sequences can specifically interact with natural proteins with functional consequences.

## Conclusions

The examples above provide substantial encouragement for the membrane protein designer. Sequences with minimal chemical diversity are chemically and biosynthetically accessible and introducing activity, for example by cofactor binding, appears to be achievable. Perhaps the major challenge with minimal proteins (just as for other *de novo* proteins) lies in understanding the particular sequence features required to arrive at a compact folded state. As put by Richardson: ‘the hardest part of protein folding, or protein design, is the last little bit' [100]. Transporters, receptors, ligand-gated channels and others exert their function through specific conformational changes; so defining explicit conformations, and the means to transition between them, will be important in any effort to recreate these functions [31]. Well-structured *de novo* membrane proteins — albeit with greater sequence complexity — have already been achieved by exploiting the helix packing properties apparent in natural proteins [30–33] and this will be supported by the continuing development of bioinformatic and computational methods [15,101]. Minimalism is a logical extension of prior work on model membrane systems that develops our essential understanding of membrane biology and could generate novel synthetic components. This makes the minimalist approach an interesting and useful stitch in the broader tapestry of protein design.

**Perspectives**

- Integral membrane proteins are very important in biological systems, but are underrepresented in *de novo* design. A design strategy that embraces strict minimalism will offer fundamental insights and could be useful for synthetic biology applications.
- Much of our current knowledge comes from studies of chemically-synthesised peptides in model bilayers. The use of naturally occurring sequence motifs to control helical packing has been a productive approach.
- Future directions should include biosynthesis and functionalisation *in vivo*, in order to integrate artificial proteins into living systems.

## Abbreviations

GFP: green fluorescent protein
PDGFβR: platelet-derived growth factor β receptor
